# Cost-effectiveness of point-of-care digital chest-x-ray in HIV patients with pulmonary mycobacterial infections in Nigeria

**DOI:** 10.1186/s12879-014-0675-0

**Published:** 2014-12-13

**Authors:** Gambo Aliyu, Samer S El-Kamary, Alash’le Abimiku, Laura Hungerford, Joshua Obasanya, William Blattner

**Affiliations:** Health and Human Services, Federal Capital Territory, Abuja, Nigeria; Department of Epidemiology and Public Health, University of Maryland School of Medicine, Baltimore, MD USA; Institute of Human Virology, University of Maryland School of Medicine, Baltimore, MD USA; National Tuberculosis and Leprosy Training Center, Zaria, Nigeria

**Keywords:** Digital chest-x-ray, HIV, Tuberculosis, Non-tuberculosis mycobacteria, Nigeria

## Abstract

**Background:**

Chest-x-ray is routinely used in the diagnosis of smear negative tuberculosis (TB). This study assesses the incremental cost per true positive test of a point-of-care digital chest-x-ray, in the diagnosis of pulmonary mycobacterial infections among HIV patients with presumed tuberculosis undetected by smear microscopy.

**Methods:**

Consecutive patients with clinical suspicion of pulmonary tuberculosis were serially tested for Human immunodeficiency virus (HIV), their sputum examined for Acid Fast Bacilli then cultured in broth and solid media. Cultures characterized as tuberculous (M.tb) and non-tuberculous (NTM) mycobacteria by Hain assays were used as gold standards. A chest-x-ray was classified as: (1) consistent for TB, (2) not consistent for TB and (3) no pathology.

**Results:**

Of the 1391 suspected cases enrolled, complete data were available for 952 (68%): 753/952 (79%) had negative smear tests while 150/753 (20%) had cultures positive for TB. Of those, 82/150 (55%) had chest-x-ray signs consistent with TB and 29/82 (35%) were positive for HIV. Within the co-infected, 9/29 (31%) had NTM infections. Among all suspects, the cost per positive case detected using smear microscopy test was $52.84; the overall incremental cost per positive case using chest-x-ray in smear negatives was $23.42, and in smear negative, HIV positive patients the cost was $15.77.

**Conclusion:**

Point-of-care chest-x-ray is a cost-effective diagnostic tool for smear negative HIV positive patients with pulmonary mycobacterial infection.

**Electronic supplementary material:**

The online version of this article (doi:10.1186/s12879-014-0675-0) contains supplementary material, which is available to authorized users.

## Background

Facilities for confirmation of tuberculosis (TB) using culture and the newer point-of-care molecular diagnostic tools are lacking in resource-limited settings. The absence of a highly sensitive, specific, simple and inexpensive tool for diagnosis of TB forces care givers in high burden settings to rely mainly on clinical and radiographic indicators to initiate treatment in smear-negative suspects [1]. Furthermore, since stationary radiographic units are only available in select hospitals, the vast majority of remotely located directly observed therapy (DOT) clinics rely on routine smear microscopy for the diagnosis and treatment of TB.

Given that non-tuberculous mycobacteria (NTM) tend to test negative for acid fast bacilli (AFB) on smear microscopy [2], and the strong association between HIV co-infection and negative AFB on smear microscopy [3]-[5], routine digital chest-x-ray may be a valuable tool for intensified detection of pulmonary TB and NTM diseases in HIV cases in settings where routine smear microscopy remains the standard of care for TB diagnosis. In these settings, HIV infected cases with clinical suspicion of smear-negative TB are routinely treated for the disease based on good radiographic signs. Chest-x-ray is the readily available tool for diagnosis of TB in smear-negative cases in these settings [6]-[8].

Smear-negative TB in HIV infected cases can be subject to diagnostic delays or incorrect diagnoses due to the absence of clinical features of TB which lead to treatment delays, inappropriate therapies and poor prognoses [1],[9]-[11]. Radiographic signs of TB in HIV-infected patients may vary significantly and can range from the absence of any radiographic sign to the classical signs such as cavitation, upper lobe infiltration [12], prominent intra-thoracic lymphadenopathy, miliary shadowing, pleural effusion, and upper lobe disease [13],[14]. The presence of one or more of the followings: upper lobe disease, cavity formation, lymphadenopathy with or without unilateral pleural effusion is reportedly associated with active TB disease in clinically symptomatic patients with greater than 80% inter-reader reliability for each of the listed signs [15].

In this paper, we examined the yield and added cost of routine digital point-of-care radiographic testing among HIV-infected patients with pulmonary mycobacterial infections in a setting with high prevalence of non-tuberculous mycobacteria using the health care provider’s perspective over a short time horizon of one year for the study implementation. While diagnostic accuracy and acceptability of this algorithm in resource-limited setting has been described [16], few studies have reported on its cost-effectiveness.

## Methods

New patients with unknown HIV status and a history of productive cough for at least three weeks were consecutively enrolled at the National TB and Leprosy Training Center (NTBLTC) in Zaria, Nigeria, from August 2010 through August 2011 as previously reported [2],[17]. Written informed consents were obtained from the patients. The study protocol was reviewed and approved by the Nigerian National Health Research Ethics Committee and the University of Maryland School of Medicine Institutional Review Board with written expression of support by the management of the NTBLTC.

HIV status was determined by the standard of care algorithm at the time of the study conduct [18] which consisted of Uni-Gold™ (Trinity Biotech PLC, Co Wicklow, Ireland) and Determine® HIV-1/2 (Abbott Laboratories. Abbott Park, Illinois, USA). Chest –x-rays were taken at enrolment using a portable digital x-ray unit: MinXray HF120/60HPPWV (MinXray, Inc. Northbrook, Illinois, USA). This direct radiographic digital imaging unit consists of an X-ray tube, imaging panel, comprehensive digital applications software, a laptop computer and wireless capability for image transmissions. This unit can be deployed to out-patient clinics, directly observed therapy (DOT) clinics within, between and outside facilities to provide radiographic imaging services to patients for a variety of purposes including surveillance [19].

Three spot sputum samples (a spot sputum sample in the clinic, then another one collected by the patient in the morning at home, and a third one the next day in the clinic [spot-morning-spot]) were collected as part of the standard of care at the time of the study. Routine direct smear examinations for acid fast bacilli (AFB) with Ziehl-Neelsen (ZN) stain were done on the two clinic collected spot samples and graded as negative if no AFB were seen in at least 100 microscopic fields of any of the two spot samples, while positive smears were graded as follows: scanty, 1-9 AFB in 100 fields; +1, 10-99 in 100 fields; +2, 1-10 per field in at least 50 fields; +3, greater than 10 per field in at least 20 fields. The early morning sputum samples, which are expected to yield high concentrations of mycobacteria, were then tested per study protocol as follows.

The samples were cultured in the automated BACTEC MGIT 960™ machine (Becton, Dickinson and Company, New Jersey, USA). Direct smear examination was not done on the early morning samples to minimize contamination. Samples without any growth after 42 days of incubation were removed and classified as negative according to the manufacturer’s protocol. Samples with growth were sub-cultured in blood agar to check for non-mycobacterial contaminations.

Positive uncontaminated cultures were tested for members of Mycobacterium tuberculosis (M.tb) complex which include: *Mycobacterium tuberculosis*, *africanum*, *bovis*, *canetti*, *microti etc*. These were tested with rapid TB antigen assay (SD-Bioline Ag MPT64 Rapid™ assay; Standard Diagnostics, Kyonggi-do, Korea) which identifies MPT64 antigen specific to members of the M.tb complex group. The M.tb complex samples were then characterized with Genotype MTBC test (Hain Lifescience, Nehren, Germany). Positive cultures with a negative test for M.tb complex on SD-Bioline were considered NTM isolates and were sub-cultured on Lowenstein Johnson (LJ) medium. Cultures with positive growth on LJ were characterized with Genotype CM/AS assay (Hain Lifescience, Nehren, Germany) the details of the mycobacteria isolation and characterization for this study are described elsewhere [2].

### Chest-x-ray diagnosis of TB

Patients had chest radiographs taken on site near the DOT clinic after providing the first spot sputum samples at the laboratory located near the clinic. All chest-x-rays were initially scored by a physician trained to read radiographic signs of TB, who was also responsible for the radiographic diagnosis of TB at the DOT clinic. The chest-x-rays were then independently scored by a visiting radiologist. Both were blinded to the patients’ smear microscopy test results. Radiographic signs like *bihilar lymphadenopathy, cavitation, localized discrete opacities, miliary pattern and pleural effusion* were considered consistent for TB (TB+) while signs like presence of solitary hilar opacity, homogenous opacities in the middle or lower lung zone, prominent vascular markings were scored as not consistent for TB (TB±). The absence of any visible pathologic sign was scored as no pathology.

### Resource cost estimation for smear microscopy and chest-x-ray

The recurrent costs of the smear and chest-x-ray diagnostic test for TB were calculated by tabulating the ingredients involved, and their individual costs were determined by: provider interview, checking the provider’s records, manufacturer interview, direct price observation and using WHO-STOP TB planning and budgeting estimates for Nigeria [20]. In evaluating the cost per test, 25 smears and 45 chest-x-ray examinations were used as the average number of tests performed daily. The economic costs of key capital investments including a MinXray machine with estimated life span of 15 years and a smear microscope equipment with estimated life span of 10 years were calculated on annualized bases at a discount rate of 3% over their estimated life spans using the WHO annualization and discount factors tables [21]. The lease value was used to estimate the cost of the facility used to provide the services and this was tabulated under the recurrent costs. The annualized value of capital items was added to the total costs of recurrent items to get the overall start-up cost. Costs initially measured in Nigerian Naira were converted to the US Dollar at the prevalent exchange rate of $1 = N152 (August, 2011).

### Statistical analysis

Measures of accuracy for the smear and chest-x-ray examinations were evaluated using the outcomes of sputum culture and Hain line probe assay as the gold standard. Kappa statistics for agreement in the radiographic diagnosis between the two interpreters were assessed and interpreted as previously suggested by Vieira and Garrett [22]. Standard tables of annualization and discount factors were used to determine the annual cost of capital items. All statistical analyses were done using statistical analysis systems (SAS) software (SAS Institute Inc, Cary, North Carolina, USA).

### Cost effectiveness analysis

In evaluating the cost effectiveness, all true culture-positive mycobacterial infections from smear or chest-x-ray tests were considered as TB cases. The unit of effectiveness was the number of TB cases correctly identified by positive chest-x-ray or smear tests. For the routine smear examination, the costs of the first and second smear tests were added then divided by the number of cases correctly identified on smear test to estimate the cost effectiveness ratio (CER) (dollars per TB case detected). The cost of chest-x-ray among the smear-negative suspects was divided by the number of TB cases among the smear-negatives identified by chest-x-ray to obtain the incremental cost-effectiveness ratio (ICER) (additional dollars per case detected). A one way sensitivity analysis was performed by varying a parameter with certain probability distribution: the proportion of NTM causing pulmonary mycobacterial infections, to evaluate changes in the ICER following digital chest radiograph given that majority were likely to remain undetected by the routine smear test. We also removed the contribution of the discounting that accounted for the opportunity cost to observe the changes in the costs per tests and the corresponding ICER values.

## Results

**Figure 1:**
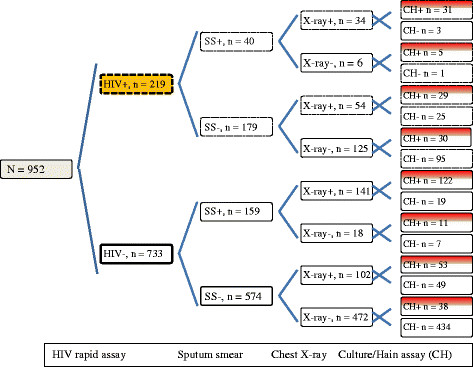
**Summary of results in the study diagnostic pathway among 952 patients with complete test outcomes: n = number of participants with a test outcome; + = positive outcome; - = negative outcome; SS = sputum smear microscopy test; X-ray = radiographic signs on chest x-ray taken with MinXray machine; CH = culture (liquid) and Hain assay (line probe assay) for the detection and differentiation of tuberculous and non-tuberculous mycobacterial species.**

A total of 1,391 patients were enrolled, with complete test results available for 952 patients (68.4%) after excluding contaminated samples from 150 patients (10.8%), and 289 patients (20.8%) who did not have a chest radiograph taken. Of the 952 patients, 753 (79%) were smear negative (SS-), 179 (23.8%) of the SS- had co-infection with HIV (HIV+) and 54 (30.2%) of the HIV + SS- had chest-x-ray signs that were considered pathologic (abnormal). Among the SS- patients who were negative for HIV (HIV-), 102 (17.8%) had chest-x-rays that were abnormal. Similarly, 199 of the 952 (20.9%) patients had a positive smear test (SS+) and within those, 40 (20.1%) were HIV+. In 34 (85%) of the HIV + SS+ the chest-x-rays were abnormal while among the SS + HIV- patients, 141 (88.7%) had abnormal chest-x-ray signs. A summary of outcome by test among the 952 patients included in this analysis is shown in Figure 1.

Of the 952 patients, 319 (34%) had true pulmonary mycobacterial infections confirmed by culture and molecular line probe assay and of those, 254 (80%) had M.tb while the remaining 65 (20%) had NTM infection. Among the 254 M.tb cases, 91 (36%) were SS-, with 35 (38%) being HIV+, and of those, 30 (86%) had abnormal chest-x-ray signs, with 20 (67%) having signs consistent with TB (Table 1). Of the 65 NTM cases identified, 59 (91%) were SS-, with 24 (43%) being HIV+, and of the SS-HIV+, 18 (75%) had abnormal chest-x-ray signs, with 9 (50%) having signs consistent with TB.

**Table 1:** **Smear microscopy and chest-x-ray findings against the gold standard among patients with complete test outcomes**

	Chest-x-ray diagnosis	Culture and Hain line probe assays					
		M.tb		NTM		Negative	
		N = 254		N = 65		N = 633	
		n	%	n	%	n	%
HIV+ SS+	TB+	30	11.8	1	1.5	3	0.5
	TB±	3	1.2	1	1.5	0	0.0
	No pathology	1	0.4	0	0.0	1	0.2
HIV+ SS-	TB+	20	7.9	9	13.9	25	3.9
	TB±	10	3.9	9	13.9	78	12.3
	No pathology	5	2.0	6	9.2	17	2.7
HIV- SS+	TB+	119	46.8	3	4.6	19	3.0
	TB±	5	2.0	0	0.0	1	0.2
	No pathology	5	2.0	1	1.5	6	0.9
HIV- SS-	TB+	38	14.9	15	23.1	49	7.7
	TB±	11	4.3	14	21.5	202	31.9
	No pathology	7	2.8	6	9.2	232	36.7

*M.tb* Mycobacterium tuberculosis complex, *NTM* Non-tuberculous mycobacterium, *SS+* Sputum smear microscopy positive, *HIV+* Human immune deficiency virus positive, *HIV-* Human immune deficiency virus negative, *TB+* Chest-x-ray signs consistent for TB, *TB ±* Chest-ray signs not consistent for TB.

However, among those SS-HIV+ whose chest-x-rays were not consistent with TB (TB±), 10 (3.9%) actually had M.tb infections, while 9 (13.9%) had NTM infections. Overall, among the 753 SS- patients, 150/753 (20%) had cultures positive for TB. Of those, 82/150 (55%) had chest-x-ray signs consistent with TB and 29/82 (35%) were positive for HIV. Within the co-infected, 9/29 (31%) had NTM infections (Table 1). When the association of HIV infection with positive chest-x-ray was tested statistically among the M.tb and NTM separately; HIV+, M.tb cases were less likely to have x-ray findings consistent with classical tuberculosis (TB+) compared to HIV-, M.tb cases: *OR =0.47, 95% CI = 0.24-0.91; P = 0.02 and Chi sqr = 5.13.* Similarly, HIV+ NTM cases were less likely to have radiographic findings consistent with the TB+ compared to HIV-, NTM cases, although this association was not found to be statistically significant: *OR = 0.87, 95% CI = 0.32-2.35, P = 0.78 and Chi sqr = 0.08.*

The levels of agreements between the two chest-x-ray readers on the three diagnostic scores were as follows: (1) consistent with TB (TB+): 82%, kappa = 0.63; (2) not consistent with TB (TB±): 75% (kappa = 0.50) and no pathology: 91% (kappa = 0.79).

### Sensitivity and specificity of direct smear for M.tb and NTM

Among the HIV+ patients, the sensitivity and specificity of the direct smear alone for M.tb infection were 49.3% (95% confidence interval (CI) = 42.7-55.9) and 81.3% (95% CI = 78.6-83.7). In contrast to M.tb, the sensitivity of the direct smear for NTM was very low at 7.7% (95% CI = 3.4-12.0) while the specificity was 78.7 (95% CI = 75.9-81.2). For the HIV- cases, the sensitivity of direct smear for M.tb and NTM were higher at 69.7% (95% CI = 66.4-73.0) and 10.3% (95% CI = 7.8-12.8) respectively while the corresponding specificities were: 90.9 (95% CI = 88.6-92.7) and 78.6 (95% CI = 75.9-81.2) (Table 2).

**Table 2:** **Sensitivity and specificity of smear microscopy and chest-x-ray for M.tb and NTM by HIV status**

		HIV positive (n = 219)		HIV negative (n = 733)	
		M.tb (n = 69)	NTM (n = 26)	M.tb (n = 185)	NTM (n = 39)
		% (95% CI)	% (95% CI)	% (95% CI)	% (95% CI)
Sputum smear test for all TB suspects	Sensitivity	49.3 [42.7-55.9]	7.7 [3.4-12.0]	69.7 [66.4-73.0]	10.3 [7.8-12.8]
	Specificity	81.3 [78.6-83.7]	78.7 [75.9-81.2]	90.9 [88.6-92.7]	78.6 [75.9-81.2]
	PPV	17.1 [12.5-22.9]	1.0 [0.3-3.6]	64.8 [58.0-71.1]	2.0 [0.8-5.1]
	NPV	95.4 [93.6-96.4]	96.8 [95.3-97.9]	92.6 [90.5-94.2]	95.4 [93.6-96.6]
Chest-x-ray for smear-negative TB suspects	Net sensitivity	78.3 [72.3-84.3]	42.3 [34.2-50.4]	90.3 [87.9-92.7]	48.7 [44.4-53.0]
	Net specificity	65.9 [62.7-68.7]	62.9 [59.7-65.9]	75.5 [72.3-78.4]	63.2 [60.0-66.3]
	Net PPV	15.2 [14-16.3]	3.1 [2.6-3.7]	47.0 [45.4-48.6]	5.4 [4.7-6.1]
	Net NPV	97.5 [96.1-98.6]	97.5 [97.1-97.8]	97.0 [96.6-97.4]	96.6 [96.1-97.0]
	Incremental yield	29.0 [22.4-35.7]	34.6 [26.8-42.4]	20.6 [17.3-23.9]	38.4 [34.2-42.6]

*M.tb* Mycobacterium tuberculosis complex, *NTM* Non-tuberculous Mycobacteria, *PPV* Positive predictive value, *NPV* Negative predictive value, *95% CI* 95% confidence interval.

When the outcomes of chest-x-ray were analyzed in sequence after the direct smear test for the *smear-negatives* (smear-chest-x-ray algorithm) the sensitivity for NTM infections increased from 7.7% to 42.3% in the HIV+ cases and from 10.3% to 48.7% among the HIV- cases. Similarly, the sensitivity for M.tb detection was raised from 49.3% to 78.3% in those with HIV and from 69.7% to 90.3% in those without HIV infection (Table 2). The positive predictive values of smear microscopy in HIV positive M.tb and NTM were 17.1 (95% CI = 12.5 -22.9) and 1.0 (0.3-3.6) respectively. For the smear-chest-x-ray algorithm, the net positive predictive values for HIV positive M.tb and NTM cases were 15.2 (14.0-16.3) and 3.1 (2.6-3.7). The predictive values were generally higher for the HIV negative cases (Table 2).

### Cost per smear and chest-x-ray examination

The cost of ingredients included in the estimation of the local cost per each test in US dollars is shown in Table 3. The TB staining kits and staff salary accounted for over 80% of the sputum smear examination cost. For the chest-x-ray, the main cost drivers were the MinXray digital imaging unit, the CD-ROMs used in reporting the chest-x-ray findings, staff salary and quality assurance. Items like furniture, detergents and all non-accounted costs were categorized as “other”. The cost of a single sputum smear examination was higher than a chest-x-ray examination.

**Table 3:** **Estimated cost of ingredients used in the performance of smear microscopy and chest-x-ray diagnostic tests**

Diagnostic test	Item	Unit	Unit cost (US dollars)	Cost per test	Source
Sputum smear (SS)	TB Stain Kit (ZN)	250 ml	138.20	2.76	Manufacturer provider’s record
	Microscope	Piece	1.73	0.03	WHO-Stop TB
	Sputum container	Piece	0.13	0.13	Provider’s record
	Applicator stick	500 Pieces	7.89	0.01	Provider’s record
	Immersion oil	500 ml	43.16	0.09	WHO-Stop TB
	Slides	50 Pieces	4.28	0.09	WHO-Stop TB
	Gloves	100 Pieces	14.47	0.15	WHO-Stop TB
	Logbook	Piece	3.90	0.02	Provider’s record
	Pen	12 Pieces	4.93	0.01	Provider’s record
	Lab coat	Per day	0.01	<0.01	WHO-Stop TB
	Staff salary	Per day	28.5	1.14	Interview
	Quality control	Per day	0.12	<0.01	Provider’s record
	Staff training	Per day	2.37	0.10	Provider’s record
	Building	Per day	0.38	0.02	Interview
	Electricity	Per day	0.75	0.03	Interview
	Water	Per day	0.30	0.01	Interview
	Other	Per day	3.50	0.14	Provider’s record
	***Cost of an SS test***			***4.73***	
Chest-x-ray (CXR)	MinXray	Package	65,000.00	0.46	Manufacturer
	Shipment	Once	1,500.00	0.01	Manufacturer
	Installation and training	Once	15,000.00	0.11	Manufacturer
	Technical support	Per year	2,500.00	0.21	Manufacturer
	Apron	Piece	100.00	<0.01	Interview
	CD-ROM	100 Pieces	0.50	0.50	Observation
	Pen	12 Pieces	4.93	0.01	Provider’s record
	Logbook	Piece	3.90	<0.01	Provider’s record
	Staff salary	Per day	32.90	0.66	Interview
	Building	Per day	0.38	<0.01	Interview
	Electricity	Per day	2.24	0.05	Interview
	Quality control	Per day	21.58	0.48	Interview
	Other	Per day	3.00	0.07	Interview
	***Cost of an CXR***			***2.56***	Oc accounted
				2.47	Oc unaccounted

*ZN* Ziehl Neelsen, *Oc* Opportunity cost for the capital investment.

### Cost-effectiveness ratio (CER) and Incremental cost-effectiveness ratio (ICER)

Among all subjects, the cost per TB case detected using an algorithm consisting of two successive sputum smear microscopy was = $52.84. When the smear tests were followed by a chest-x-ray in subjects with SS- results regardless of their HIV status, the incremental cost per additional case identified was = $23.42. However, the incremental cost decreased to = $15.77 when chest-x-ray was limited to the SS-HIV+ patients, accounting for the opportunity cost in the capital items of chest-x-ray machine and its accessories, and smear microscope. When the costing was done without the opportunity cost, the cost per chest-x-ray dropped from $2.56 to $2.47 with the corresponding changes in the ICER summarized in Table 4. The cost per smear microscopy test virtually remains unchanged ($4.73 to $4.74) thus no changes in the CER were seen. When the sensitivity analysis was done by altering the prevalence of NTM infection among the cases above and below the 20% findings in this study; the ICER of the smear-chest-x-ray algorithm decreased with increase in prevalence and vice-versa. At a low prevalence of 5% the ICER = $20.8 for SS-HIV+ (Table 5).

**Table 4:** **Cost per case detected on SS and incremental cost-effectiveness ratio (ICER) for additional case detected on chest-x-ray**

*Test positive*	*Group*	*TB+ cases*	*% NTM*	*CER*	*ICER*	
					With Oc	Without Oc
Sputum smear	All	169	3.6	$52.84		
Sputum smear	HIV+	36	5.6	$57.06		
Sputum smear	HIV-	133	3.0	$51.70		
Chest-x-ray	SS-	82	29.3		$23.42	$22.68
Chest-x-ray	SS-HIV+	29	31.0		$15.77	$15.25
Chest-x-ray	SS-HIV-	53	28.3		$27.67	$25.49

*SS-* Sputum smear negative, *TB+* Tuberculosis positive, *NTM* Non tuberculous mycobacteria, *HIV+* Human immune deficiency virus positive, *CER* Cost effectiveness ratio, *Oc* Opportunity cost.

**Table 5:** **Changes in the incremental cost-effectiveness ratio (ICER) with changes in the prevalence of pulmonary NTM infections**

	*Smear negative all*		*Smear negative HIV+*	
Prevalence NTM	TB+	ICER (US $)	TB+	ICER (US $)
35%	99	19.45	36	12.7
30%	93	20.70	34	13.5
25%	88	21.90	31	14.8
20%	82	23.50	29	15.8
15%	76	25.36	27	17.0
10%	70	27.54	25	18.3
5%	64	30.12	22	20.8

*NTM* Non tuberculosis mycobacteria, *TB+* smear negative cases with chest-x-ray signs consistent with tuberculosis.

## Discussion

The outcome of this study shows that chest-x-ray in smear-negative TB suspects could significantly improve the yield of mycobacterial chest infections undetected by routine smear tests. The previously reported low sensitivity of smear microscopy test among HIV positive is further confirmed by our findings [23],[24]. The sensitivity is lowest in HIV positive cases with NTM infections. However, the cost for detecting cases among the smear-negatives with chest-x-ray signs that are consistent for TB is lowest in those with HIV co-infection. This could be attributed to the higher than expected yield of radiographic signs among HIV patients with NTM infections and the numerical differences between HIV infected and uninfected in which the infected are less. The high yield of the smear-chest-x-ray algorithm for NTMs among the smear-negatives means additional cases of the disease with radiographic signs could be detected at less cost when compared to the rate at which these cases are initially diagnosed by smear microscopy alone.

The reports on the performance of smear-chest-x-ray algorithm in resource-limited settings with a high prevalence of HIV show in one study, the algorithm correctly identified more than half of the smear-negative TB-HIV cases while in another, less than half of the cases are identified [16],[25]. Although GeneXpert MTB/RIF is endorsed by WHO for routine screening of TB in HIV+ [26],[27], the test however, cannot detect NTMs [28], and given the observed low sensitivity of smear test for the NTMs; chest-x-ray examination then becomes crucial in settings where NTM is prevalent and diagnostic test options are limited, or among those with clinical features of the disease who test negative on GeneXpert.

Smear-negative TB is a public health concern due to the attributably high morbidity and mortality in people living with HIV and acquired immune deficiency syndrome (AIDS) in resource-limited settings [9],[29]. Interestingly, point-of-care chest-x-ray requires less than half of the resources needed for the initial smear microscopy diagnostic tests. Although identifying these cases is crucial, treating them may not be that straightforward since the response of NTM to conventional TB drugs in this setting is not known yet.

In this study, false positive cases are identified because of the culture and molecular line probe assays which are not part of the standard of care in Nigeria. In practice, when chest-x-ray is performed on smear-negative presumed cases of TB, the false-positives also receive treatment for TB. Some have used the initial response to antibiotics like amoxicillin to differentiate TB from non-TB pulmonary infections [24],[30]. The trade-off between compromising specificity and positive predictive in favor of sensitivity comes to play whenever the goal of multiple diagnostic tests is to maximize disease detection. The cost implication of treating HIV cases with false-positive TB has to be taken into consideration when sensitivity is valued over specificity, since the false positives can be as many as the true-positives. In HIV patients with presumed TB, the public health implication of missing a true case of TB far outweighs the added cost of detecting and treating a false case of TB. In this study, a cheap second diagnostic test minimizes false-negative TB by picking all patients with abnormal chest-x-ray notwithstanding the few HIV infection cases that may present with a normal chest-x-ray as our findings show as well as previous reports [10],[31]. The HIV co-infected cases also have the tendency to present with radiographic findings which are not consistent with classical tuberculosis. This is consistent with the reported findings of Kisembo et al. [32] in which chest radiographs of HIV negative tuberculosis cases showed more consolidations and cavities compared to those of HIV positive tuberculosis cases.

In the absence of a more efficient, cheap and scalable diagnostic tool for NTM in resource-limited settings, point-of-care digital chest-x-ray in HIV patients with smear-negative results may significantly improve diagnosis and treatment of TB. Given that the machine is portable, it can be shared between different sites which could further lower the cost and improve access to care. The device operates on portable power generators and is physically smaller; it requires less space without heavy maintenance and unlike the conventional stationary X-ray machines it can fit into different DOT clinic settings. While the initial capital investment covering the machine cost, shipment, transportation and installation is significant (about $81,500 U.S dollars), when spread over the estimated 15 years life time of the machine, we still found its use to be cost-effective.

## Conclusions

In conclusion, the incremental cost of identifying smear negative HIV positive TB cases with radiographic signs of tuberculosis using point-of-care digital chest-x-ray is about 30% of the cost of the initial smear microscopy test. Given that culture and molecular line probe assays are not scalable in these settings and GeneX-pert cannot identify NTM infections, making the point-of-care digital chest-x-ray routine for smear negatives in all DOT sites will also enhance detection and treatment of pulmonary non-tuberculosis mycobacterial infections and maximize the yield of tuberculosis cases in HIV positive patients.

## Authors’ original submitted files for images

Below are the links to the authors’ original submitted files for images. Authors’ original file for figure 1

## Abbreviations

HIV: Human immune deficiency virus
AFB: Acid fast bacilli
M.tb: Mycobacterium tuberculosis
NTM: Non-tuberculous mycobacteria
DOT: Directly observed therapy
NTBLTC: National tuberculosis and leprosy training center
ZN: Ziehl Neelsen
MGIT: Mycobacteria growth indicator tube
LJ: Lowenstein Johnson
TB+: Chest-x-ray signs consistent for tuberculosis
TB±: Chest-x-ray signs not consistent for tuberculosis
HIV+: Human immune deficiency virus positive
HIV-: Human immune deficiency virus negative
SS+: Sputum smear positive
SS-: Sputum smear negative
CER: Cost-effectiveness ratio
ICER: Incremental cost-effectiveness ratio
