# Supplementary figures and images for: Cost-effectiveness of point-of-care digital chest-x-ray in HIV patients with pulmonary mycobacterial infections in Nigeria
Source: BMC Infect Dis. 2014 Dec 13;14:675. doi: 10.1186/s12879-014-0675-0 (PMC4269933; doi:10.1186/s12879-014-0675-0)

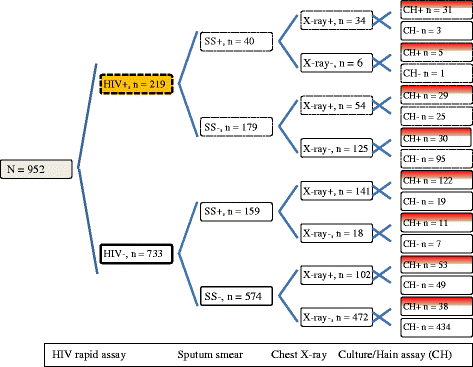

Supplement: Supplementary file 1 — Authors’ original file for figure 1 [file 12879_2014_675_MOESM1_ESM.gif]
